# *Bacillus subtilis* Colonization of *Arabidopsis thaliana* Roots Induces Multiple Biosynthetic Clusters for Antibiotic Production

**DOI:** 10.3389/fcimb.2021.722778

**Published:** 2021-09-03

**Authors:** Harsh Maan, Omri Gilhar, Ziv Porat, Ilana Kolodkin-Gal

**Affiliations:** ^1^Department of Molecular Genetics, Weizmann Institute of Science, Rehovot, Israel; ^2^Flow Cytometry Unit, Life Sciences Core Facilities, Weizmann Institute of Science, Rehovot, Israel

**Keywords:** flow cytometry, imaging, antibiotics, *Bacillus*, plant

## Abstract

Beneficial and probiotic bacteria play an important role in conferring immunity of their hosts to a wide range of bacterial, viral, and fungal diseases. *Bacillus subtilis* is a Gram-positive bacterium that protects the plant from various pathogens due to its capacity to produce an extensive repertoire of antibiotics. At the same time, the plant microbiome is a highly competitive niche, with multiple microbial species competing for space and resources, a competition that can be determined by the antagonistic potential of each microbiome member. Therefore, regulating antibiotic production in the rhizosphere is of great importance for the elimination of pathogens and establishing beneficial host-associated communities. In this work, we used *B. subtilis* as a model to investigate the role of plant colonization in antibiotic production. Flow cytometry and imaging flow cytometry (IFC) analysis supported the notion that *Arabidopsis thaliana* specifically induced the transcription of the biosynthetic clusters for the non-ribosomal peptides surfactin, bacilysin, plipastatin, and the polyketide bacillaene. IFC was more robust in quantifying the inducing effects of *A. thaliana*, considering the overall heterogeneity of the population. Our results highlight IFC as a useful tool to study the effect of association with a plant host on bacterial gene expression. Furthermore, the common regulation of multiple biosynthetic clusters for antibiotic production by the plant can be translated to improve the performance and competitiveness of beneficial members of the plant microbiome.

## Introduction

Rhizobacteria can promote plant growth directly by colonization of the root and exert beneficial effects on plant growth and development (Kloepper et al., 2004). These bacteria are often designated plant growth-promoting rhizobacteria (PGPR). To date, various PGPR have been isolated, including various *Bacillus* species, *Burkholderia cepacia*, and *Pseudomonas fluorescens.* These beneficial rhizobacteria can also confer fitness on their hosts by activating their immune system and by antagonizing plant pathogens (Berg et al., 2017; Berg and Raaijmakers, 2018; Allaband et al., 2019). In addition to the direct promotion of plant growth, PGPR enhance the efficiency of fertilizers and aid in degrading xenobiotic compounds (Adam et al., 2016; Berg et al., 2017).

Among growth-promoting strains and biocontrol agents, *Bacillus subtilis* and *subtilis* clade members, such as *B. atrophaeus, B. velezensis*, and *B. mojavensis*, are considered model organisms (Fan et al., 2017). In particular, the antimicrobial activity of *B. subtilis* has so far been demonstrated against bacterial, viral, and fungal soil-borne plant pathogens (Kloepper et al., 2004; Nagorska et al., 2007). This activity is mediated largely by antibiotic production: approximately 5% of the *B. subtilis* genome is dedicated to the synthesis of antimicrobial molecules by non-ribosomal peptide synthetases (NRPSs) or polyketide synthases (PKSs) (Stein, 2005; Ongena and Jacques, 2008; Kinsella et al., 2009; Caulier et al., 2019). *In vitro* and *in planta* studies have indicated the importance of four antibiotics for plant protection: surfactin, bacilysin, plipastatin, and bacillaene (Stein, 2005; Hou and Kolodkin-Gal, 2020; Arnaouteli et al., 2021; Ngalimat et al., 2021).

Surfactin is a small cyclic lipopeptide induced during the development of genetic competence (Magnuson et al., 1994). The machinery for surfactin synthesis is encoded within the *srfAA–AB–AC–AD* operon (Kluge et al., 1988). Surfactin is a powerful surfactant with antibacterial (Gonzalez et al., 2011) and antifungal properties (Falardeau et al., 2013). The expression of *srfAA-AD* operon is induced by the ComQXPA quorum-sensing system at the end of the exponential growth phase. In response to the competence pheromones, the phosphorylated response regulator ComA~P activates the transcription of the *srf* operon (Roggiani and Dubnau, 1993; Auchtung et al., 2006). A recent study reported that interaction with rice seedlings induces the expression on *srfAA* in *B. subtilis* OKB105 (Xie et al., 2015).

Bacilysin is a non-ribosomal dipeptide composed of L-alanine and amino acid L-anticapsin (Hernandez-Valdes et al., 2020), which demonstrates antibacterial activity against a wide range of pathogens (Walker and Abraham, 1970; Chen et al., 2009; Hou and Kolodkin-Gal, 2020). Its synthesis is controlled mainly by the *bac* operon (*bacABCDE)* (Inaoka et al., 2003; Rajavel et al., 2009).

Fengycin/plipastatin is a lipopeptide comprising 10 amino acid core linked to a β-hydroxy fatty acid and is synthetized by five plipastatin synthetases (*ppsA, ppsB, ppsC, ppsD*, and *ppsE*) (Tsuge et al., 2007). The *ppsA-E* operon is repressed by the transition state regulator AbrB during the exponential growth phase and is induced in the stationary phase. The pleiotropic regulator *degQ* gene increases the transcription from the plipastatin promoter (Rajavel et al., 2009).

Bacillaene and dihydrobacillaene (Butcher et al., 2007; Straight et al., 2007) are polyketides synthesized by an enzymatic complex encoded in the *pks* gene cluster (Butcher et al., 2007; Straight et al., 2007). The 5′ UTR of *pksC* was found to be an element for induction of the *pks* operon. The transcription of the biosynthetic clusters for bacillaene is primarily controlled by the transition phase regulators Spo0A and CodY and is also affected by DegU, ComA, and ScoC (Vargas-Bautista et al., 2014). Interestingly, we recently found that the plant host can enhance the efficiency of the killing of *Serratia plymuthica* by *B. subtilis* by inducing the synthesis of the antibiotic bacillaene (Ogran et al., 2019). These preliminary results raise the question on whether additional antibiotics that contribute to rhizocompatibility are induced by *Arabidopsis thaliana* to promote the colonization of preferred symbionts. However, as the transcriptional regulation of the biosynthetic clusters for antibiotics is diverse, this hypothesis needs to be evaluated experimentally.

To address this question systematically, we considered the overall effect of the plant host in regulating the transcription from four distinct promoters for *B. subtilis* antibiotics: surfactin, bacillaene, bacilysin, and plipastatin. As the population within *B. subtilis* biofilms and root associated communities is heterogeneous (Lopez et al., 2009; Beauregard et al., 2013; Tian et al., 2021), we monitored the expression in the single-cell level relying on flow cytometry and imaging flow cytometry (IFC). The latter combines the power and speed of traditional flow cytometers with the resolution of a microscope. It therefore allows for high rate complex morphometric measurements in a phenotypically defined way (Zuba-Surma et al., 2007). Our results indicated that the attachment with the root can specifically enhance antibiotic production and therefore may affect the competitiveness of root-associated bacteria compared with their free-living counterparts.

The use of IFC to study gene expression in bacteria was shown in several studies, as, for example, a study that examined the promoter activity of various genes *in E. coli* during the lag phase (Madar et al., 2013). However, to the best of our knowledge, this study is the first time this system has been used to study plant–bacteria interactions and the impact of such interactions on production of all four NRPs/PKS antibiotics.

## Results

We wanted to investigate whether the plant can affect the production of several NRPs/PKS antibiotics and to compare between the different plant influences. Hence, we generated *B. subtilis* strains harboring transcriptional fusions for P*_srfAA_*-yfp (surfactin), P*_pksC_*-gfp (bacillaene), P*_bacA_*-gfp (bacilysin), and P*_ppsA_*-gfp (plipastatin) (**Table 1**). The native promoters of these antibiotics regulate transcription of NRPs/PKS and the rate of transcription is influenced by binding of specific transcription factors.

**Table 1:** Common and distinct regulation of NRPS-PKS biosynthetic gene clusters.

NRPs/PKS	Promoter	Regulation (Transcription Factor)
Surfactin	P*_srfAA_* (Nakano et al., 1991)	CodY (Brinsmade, 2017), ComA (Kunst et al., 1994), ComK (Kunst et al., 1994), SigD (Allmansberger, 1997), and SigW (Haldenwang, 1995)
Bacillaene	P*_pksC_* (Vargas-Bautista et al., 2014)	ComK (Kunst et al., 1994), ScoC (Kallio et al., 1991), TnrA (Tojo et al., 2005), PerR (Herbig and Helmann, 2001), Fur (Fuangthong et al., 2002), CodY (Brinsmade, 2017), SigW (Haldenwang, 1995), SigX (Ogura and Asai, 2016), and DegU (Kunst et al., 1994)
Bacilysin	P*_bacA_* (Mariappan et al., 2012)	ComK (Kunst et al., 1994), AbrB (Strauch et al., 2007), DegU (Kunst et al., 1994), SigH (Saujet et al., 2011), and SigA (Haldenwang, 1995)
Plipastatin	P*_ppsA_* (Yaseen et al., 2016)	ComK (Kunst et al., 1994), AbrB (Strauch et al., 2007), Xre (Wood et al., 1990), DegU (Kunst et al., 1994), SigE (Haldenwang, 1995), and SigA (Haldenwang, 1995).

This table indicates the common and distinct putative transcription factors that potentially bind to the native promoter region of each operon. Transcriptional regulation analysis was performed using DBTBS Database (https://dbtbs.hgc.jp/) at a threshold: p-value 0.05. References refer to the transcription factors and their diverse roles in B. subtilis physiology.

**Figure 1:**
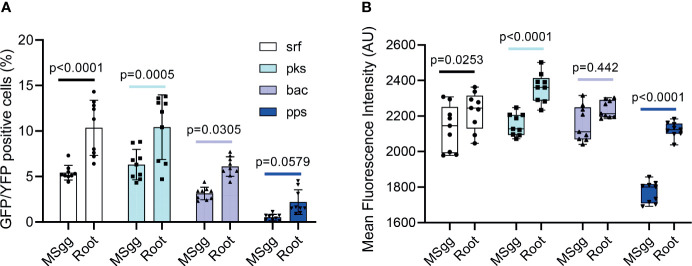
The indicated *B. subtilis* strain harboring P*_srfAA_*_-_yfp (surfactin), P*_pksC_*_-_gfp (bacillaene), P*_bacA_*_-_gfp (bacilysin), and P*_ppsA_*_-_gfp (plipastatin) reporters was analyzed by flow cytometry for **(A)** positively expressing fluorescent populations. Graphs represent mean ± SD. **(B)** The mean intensity of the fluorescent populations. Box and whisker plot shows median and interquartile range, together with the maximum and minimum values and outlier points. *B. subtilis* reporter strains were grown in either MSgg medium or MSgg medium in the presence of *A. thaliana* seedlings. Data were collected from 24 h post inoculation, and 100,000 cells were counted. Graphs represent results from three independent experiments with *n* = 3/experiment (total *n* = 9/group). Statistical analysis was performed using two-way ANOVA followed by Tukey’s multiple comparison *post hoc* testing. *p* < 0.05 was considered statistically significant.

First, we asked whether the association of *B. subtilis* with the plant root impacts the expression of each of the antibiotics. Hence, using flow cytometry, we examined the expression of transcriptional reporters by comparing bacteria cultured in liquid MSgg medium with bacteria attached to plant roots. In order to select the bacteria attached to plant roots, the plant roots were washed to remove non-adherent cells. Our flow cytometry analysis showed that the percentage of cells expressing the fluorescent reporter of each operon significantly increased after root attachment in *srf* (*p* < 0.0001), *pks* (*p* < 0.0005), and *bac* (*p* = 0.03) promoters, while it was not significantly increased in *pps* (*p* = 0.579). In addition, the mean intensity of cells expressing the promoters increased significantly in *pps* (*p* < 0.0001) and *pks* (*p* < 0.0001) (**Figure 1**).

This conventional flow cytometry allows accurate high-throughput quantification of fluorescence intensities; however, it is less accurate for bacterial analysis. Higher fluorescence levels may be interpreted as higher expression levels, but also can result from larger bacterial size or small aggregates (Branda et al., 2001; Vlamakis et al., 2008; Chai et al., 2010). Furthermore, the mean intensities measured were indicators of the entire fluorescent positive population rather than an individual bacterial cell. Therefore, to increase our resolution into the manner by which antibiotic promoters respond to the attachment of the root, we used IFC. By collecting large numbers of digital images per sample and providing a numerical representation of image-based features, the ImageStream^X^ Mark II combines the per-cell information content provided by standard microscopy with the statistical significance afforded by large sample sizes common to traditional flow cytometry.

**Figure 2:**
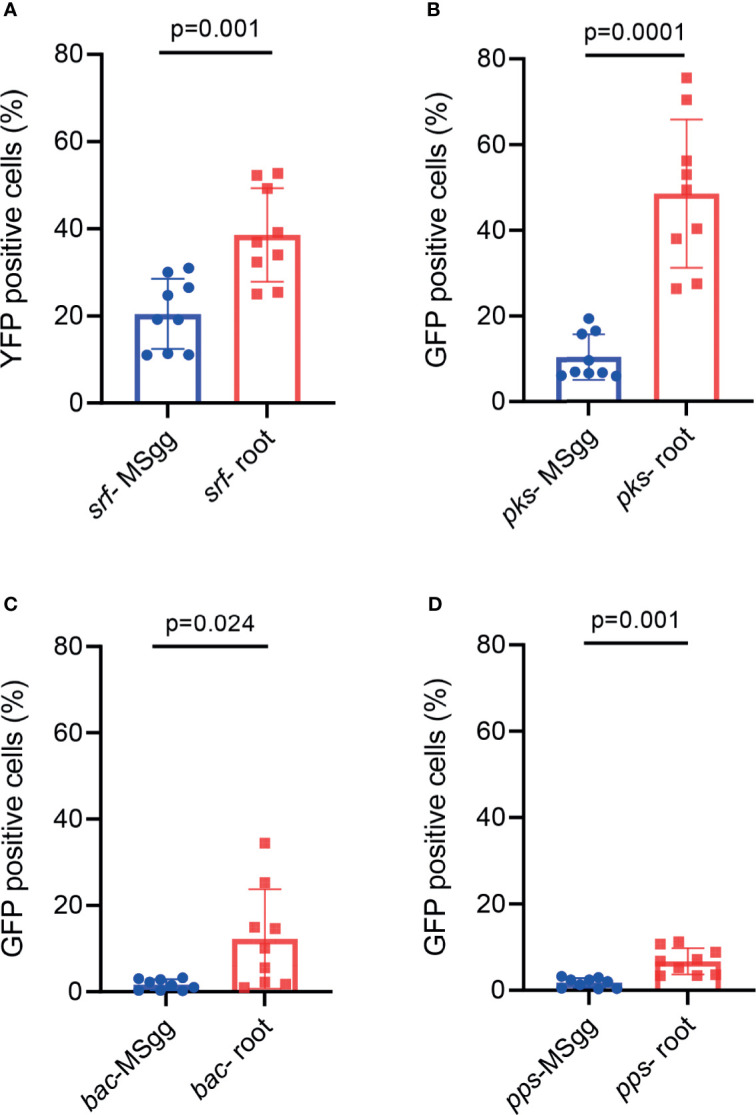
The indicated *B. subtilis* strain harboring **(A)** P*_srfAA_*_-_yfp (surfactin), **(B)** P*_pksC_*_-_gfp (bacillaene), **(C)** P*_bacA_*_-_gfp (bacilysin), and **(D)** P*_ppsA_*_-_gfp (plipastatin) reporters was analyzed by imaging flow cytometry for positively expressing fluorescent populations. *B. subtilis* reporter strains were grown in either MSgg medium or MSgg medium in the presence of *A. thaliana* seedlings. Data were collected from 24 h post inoculation, and 100,000 cells were counted. Graphs represent results from three independent experiments with *n* = 3/experiment (total *n* = 9/group). The percentage of fluorescent positive cells was measured by imaging flow cytometry and analyzed with IDEAS 6.3. Statistical analysis was performed using unpaired *t*-test with Welch correction. *p* < 0.05 was considered statistically significant. Graphs represent mean ± SD.

**Figure 3:**
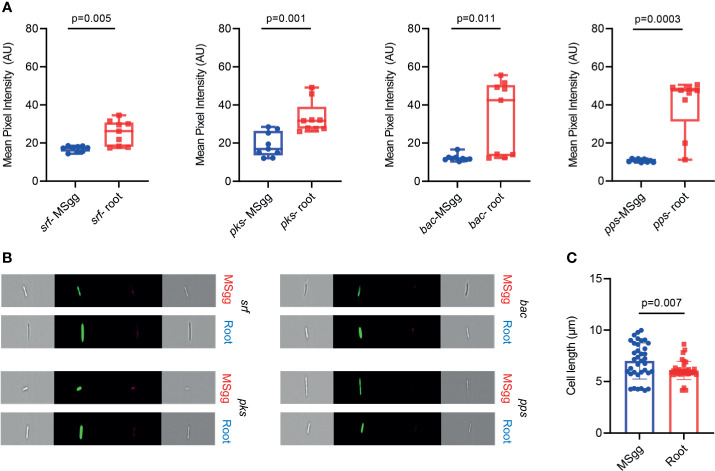
The indicated *B. subtilis* strain harboring P*_srfAA_*_-_yfp (surfactin), P*_pksC_*_-_gfp (bacillaene), P*_bacA_*_-_gfp (bacilysin), and P*_ppsA_*_-_gfp (plipastatin) reporters was analyzed by imaging flow cytometry for **(A)** mean pixel intensity of the fluorescent positive populations. Bacteria expressing the fluorescent reporters were cultured in the absence or presence of *A. thaliana* seedlings. Box and whisker plot shows median and interquartile range, together with the maximum and minimum values and outlier points. **(B)** Representative bright-field and fluorescent images related to expression of reporters in MSgg and on *A. thaliana* roots. Scale bar = 7 µm. **(C)** Comparison between cell length of all four reporters when grown in MSgg medium and cell length of the same reporters grown on *A. thaliana* roots. Graphs represent mean ± SD. Data were collected from 24 h post inoculation, and 100,000 cells were counted. The mean pixel intensity and cell length of fluorescent positive cells were measured by imaging flow cytometry and analyzed with IDEAS 6.3. Graphs represent results from three independent experiments with *n* = 3/experiment (total *n* = 9/group). Statistical analysis was performed using unpaired *t*-test with Welch correction. *p* < 0.05 was considered statistically significant.

This analysis indeed allowed us to exclude most of the bacterial doublets or small aggregates and calculate more accurately both the percentage of positive GFP cells and bacterial cell length and GFP intensity normalized for cell size. This detailed analysis of antibiotic reporters demonstrated that the percentage of fluorescent positive cells for all four antibiotics increased when attached to the plant roots (**Figure 2**). Interestingly, when attached to plant roots, percentage of cells from P*_srfAA_*-yfp (surfactin) showed an increase of ≈2-fold, P*_pksC_*-gfp (bacillaene) showed an increase of ≈5-fold, P*_bacA_*-gfp (bacilysin) showed an increase of ≈10-fold, and P*_ppsA_*-gfp (plipastatin) showed an increase of ≈3-fold. Such clear differences were not observed in the conventional flow cytometry. Furthermore, the mean fluorescent pixel intensity of the cells significantly increased following root attachment in all four antibiotic promoters (**Figures 3A, B**).

Interestingly conventional flow cytometry could not explore differences in the mean fluorescence intensity of cells from P*_srfAA_*-yfp (surfactin) and P*_bacA_*-gfp (bacilysin) when attached to roots (**Figure 1**) compared with cells grown in MSgg medium (*p* = 0.253 and *p* = 0.442, respectively). However, IFC indicated that the mean pixel intensity of the fluorescent populations of these two reporters was significantly higher (*p* = 0.005 and *p* = 0.011, respectively), showing a higher sensitivity of the method.

The quantification performed with IFC also yielded a higher percentage of fluorescence positive cells as compared with the traditional flow cytometry in all four promoters in the presence of plant root. This may be explained by the sensitivity and ability to acquire individual pixels by IFC, allowing the detection of low fluorescence intensity signals of small objects that might be within the electronic noise of conventional flow cytometers, as well as elimination of debris and aggregates. This results in better identification and separation of the bacterial population from the background noise and often provides more robust results. Overall, these results suggest that IFC is a sensitive and accurate technique to study weakly expressed genes in bacterial cells. Importantly, both flow cytometry and IFC agreed that all four antibiotics are induced by the plant.

Next, we examined if the plant and its secretions specifically regulate antibiotic production or induce all genes in *B. subtilis* due to non-specific effect of its growth, as the plant may affect the synthesis or stability of GFP regardless of its promoter. For this purpose, we measured the expression of transcriptional reporter of the β-lactamase PenP. β-lactamases are enzymes that account for an additional layer of defense as they hydrolyze the β-lactam ring of β-lactams, thus inactivating the antibiotic before it reaches its target, the PBPs (penicillin binding proteins) (Therrien and Levesque, 2000). The active β-lactamase of *B. subtilis* (Takagi et al., 1993; Bucher et al., 2019) was not induced but rather modestly decreased (*p* = 0.015) in the presence of the plant (**Supplementary Figure S1**). In addition, the expression of the core metabolic enzyme lactate dehydrogenase *ldh* was also unaffected by the root (**Supplementary Figure S1**). Therefore, the effect of the plant is not through post-translational effects on the GFP reporter, and at least two enzymes unrelated to the biosynthesis of NRP/polyketide antibiotics are not induced by the plant.

To study whether attachment to plant roots also influences bacterial cell length, we grouped all measurements into two groups, bacteria grown in MSgg medium (control) and bacteria attached to plant roots. Interestingly, when we compared the cell length among the bacteria from all the reporters attached to plant roots to that of MSgg, the data in the MSgg group showed a higher degree of dispersion (SD =1.76) as compared to the bacteria attached to the plant roots (SD = 0.90). An *F*-test further confirmed that the variances were significantly different between two groups (*F*-test, *p*-value = 0.0001) (**Figure 3C**).

**Figure 4:**
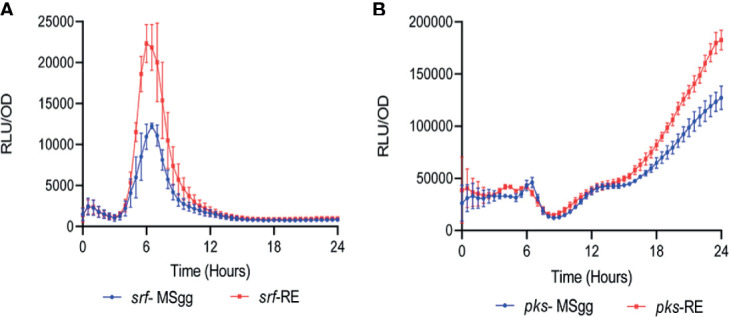
*A. thaliana* secretions increase the expression of **(A)** P*_srfAA_*_-_lux (surfactin) and **(B)** P*_pksC_*_-_lux (bacilllane) in *B. subtilis* cells. *B. subtilis* strains expressing luciferase under the control of the *srf* and *pks* promoters were cultured in liquid MSgg medium or liquid MSgg medium supplemented with *A. thaliana* secretions (15% v/v). Luminescence was monitored for 24 h using a microplate reader. Graphs represent results from three independent experiments. Error bars represent ± SEM. Luciferase activity was normalized to avoid artifacts related to differential cell numbers as RLU/OD.

**Figure 5:**
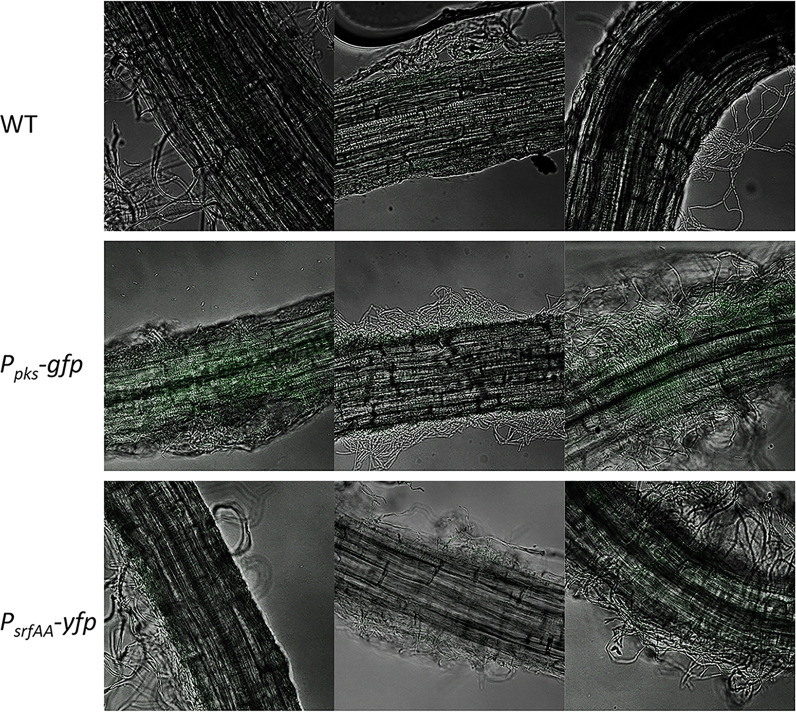
Visualizing the expression of P*_pksC_*_-_gfp (bacillaene) and P*_srfAA_*_-_gfp (surfactin) on *A. thaliana* roots. Bacteria expressing GFP under the control of the *pks* and *srf* promoters were cultured in the presence of *A. thaliana* seedlings in MSgg medium. After 24 h, the bacteria colonizing the roots were photographed with a confocal microscope.

To confirm that the impact of the plant root on antibiotic production could be due to a secreted factor, we monitored the expression from P*_srfAA_* and P*_pksC_* fused to a luciferase reporter in the presence and absence of root secretions. The use of the unstable luciferase (an enzyme that degrades rapidly and therefore is not accumulated) (McLoon et al., 2011) as a reporter allows us to monitor gene expression in real time by monitoring light production in a plate reader with an illuminometer. When grown on liquid defined medium, wild-type cells expressed luciferase from *srfAA* and *pksC* promoters robustly. However, while root secretions did not alter the kinetics of the expression, they were sufficient to significantly increase the intensity of the luciferase emission (**Figure 4**). Therefore, the measurement of the inducing effect on the level of the population agreed with our single-cell analysis and confirmed an induction by *A. thaliana* and also suggested that this induction is mediated by a secreted factor that can activate the distinct promoters for bacillaene and surfactin synthesis (**Table 1**). Consistently, using confocal scanning laser microscopy, we could clearly confirm the expression of the *pks* promoter and, to some extent, surfactin on cells attached with *A. thaliana* roots (**Figure 5**). This experiment also exemplified the adherence of the bacteria to the plant root after 24 h of co-culture, which was also the time point used to quantify the expression from the four promoters of the antibiotic biosynthetic clusters by flow cytometry and IFC.

## Discussion

Since *B. subtilis* was first described by Ferdinand Cohn in the late 1800s, it was shown to specialize in the production of secondary metabolites (Steinke et al., 2021). Many of the biosynthetic pathways for these metabolites are conserved either across the entire *Bacillus* genus or within specific phylogenetic clades. Surfactin, bacillaene, bacilysin, and plipastatin have essentially been observed within the *subtilis* clade (Aleti et al., 2015; Hou and Kolodkin-Gal, 2020). Therefore, the different environmental niches inhabited by members of the *B. subtilis* clade may select for conservation of metabolites with distinct (or potentially redundant) beneficial functions.

Here, we found that the production of non-ribosomal peptides and polyketides was specifically activated during symbiotic interaction with *A. thaliana*. Our results demonstrated that direct interactions with the root increased the expression of four different biosynthetic clusters with distinct promoters encoding for antibiotics with significance for *B. subtilis* competitiveness, and the secretions were sufficient to induce surfactin and bacillaene expression. The four promoters of the biosynthetic clusters differ in their sequence and regulations (**Table 1**). Therefore, it is not intuitive that they will be co-induced together following attachment to the root.

Indeed, various studies reported that plant metabolites induce the expression of NRPs and antibiotic production genes. We previously demonstrated that the interaction with the plant increases the capacity of *B. subtilis* to compete with *Serattia plymuthica*, and our current results further indicate that the root is an active regulator of the competitive interactions occurring on its roots (Ogran et al., 2019). An increase in bacterial competitiveness due to increased antibiotic production may be a conserved feature of rhizobacteria: plants enhance the killing efficiency of *Xanthomonas citri* by *Paenibacillus polymyxa* SC2 (Liu et al., 2021), wheat extract induces the expression of biosynthetic genes for antibiotic production in *Pseudomonas* genotypes (Rieusset et al., 2021), and barley induces the antifungal genes of *Pseudomonas fluorescens* (Jousset et al., 2011). Our methodology here offers a practical approach to study the effect of plant metabolites on heterogeneous communities, even when expressed by a small subpopulation of cells, with IFC to analyze the population at the single-cell level, and luciferase-based reporters to screen for potential activators. While flow cytometry can detect the trends efficiently, statistical significance is frequently not achieved for antibiotic producers while performing multiple comparisons. These cases include weakly expressing promoters and heterogeneous populations.

Our findings that all four biosynthetic clusters were induced by the root strongly indicate co-evolution of the regulation of biosynthetic clusters for antibiotic production. The complexity of these antibiotic–host interactions suggests that the plant host actively promotes the establishment of the most beneficial bacterial community. Our findings provide a simple example of high-order interactions that shape microbiomes; the host modulates antibiotic production in the desired bacterial colonizers, providing the colonizers a clear advantage over less beneficial potential residents.

## Materials and Methods

### Strains and Media

All strains used in this study are in **Table 2**. The strains were grown in LB broth (Difco) or MSgg medium (Branda et al., 2001) [5 mM potassium phosphate, 100 mM MOPS (pH 7), 2 mM MgCl_2_, 50 µM MnCl_2_, 50 µM FeCl_3_, 700 µM CaCl_2_, 1 µM ZnCl_2_, 2 µM thiamine, 0.5% glycerol, 0.5% glutamate, 50 µg/ml threonine, tryptophan, and phenylalanine) (Branda et al., 2001]. Solid LB medium contained 1.5% bacto agar (Difco).

**Table 2:** Strains used for this work.

Strain	Description	Source or reference
B. subtilis	Wild type	(Branda et al., 2001)
P_pksC_-lux	B. subtilis sacA::P_pksC_-lux (Cm ^r^), promoter of bacillaene operon tagged to the luciferase reporter integrated in the neutral SacA locus	(Ogran et al., 2019)
P_srfAA_-lux	B. subtilis sacA::P_srfAA_-lux (Cm ^r^), promoter of surfactin operon tagged to the luciferase reporter integrated in the neutral sacA locus	(Maan et al., 2021)
P_srfAA_-yfp	B. subtilis sacA:: P_srfAA_-yfp (Sp ^r^), promoter of surfactin operon tagged to the YFP reporter integrated in the neutral amyE locus	Avigdor Eldar Lab, TAU, Israel
P_pksC_-gfp	B. subtilis amyE:: P_srfAA_-yfp (Cm ^r^), promoter of bacillaene operon tagged to the GFP reporter integrated in the neutral amyE locus	(Ogran et al., 2019)
P_bacA_-gfp	B. subtilis amyE:: P_bacA_-gfp (Cm ^r^), promoter of bacilysin operon tagged to the GFP reporter integrated in the neutral amyE locus	(Maan et al., 2021)
P_ppsA_-gfp	B. subtilis amyE:: P_ppsA_-gfp (Cm ^r^), promoter of plipastatin operon tagged to the GFP reporter integrated in the neutral amyE locus	(Maan et al., 2021)
P_penP_-gfp	B. subtilis amyE:: P_penP_-gfp (Sp ^r^), promoter of plipastatin operon tagged to the GFP reporter integrated in the neutral amyE locus	(Bucher et al., 2019)
P_ldh-_gfp	B. subtilis amyE:: P_ldh_-gfp	(Hassanov et al., 2018)

Cm ^r^, chloramphenicol resistance; Sp ^r^, spectinomycin resistance.

### Plant Growth Conditions

Seeds of *A. thaliana* Col-0 were surface-sterilized and seeded on petri dishes containing Murashige and Skoog medium (4.4 g/L), pH 5.7, supplied with 0.5% (w/v) plant agar (Duchefa) and 0.5% sucrose (Sigma-Aldrich), and then stratified at 4°C for 2 days. The seeds were further transferred to a growth chamber (MRC) at 23°C in a 12-h light/12-h dark regime.

### Extraction of Plant Secretions

Plant secretions were retrieved from 14-day-old *A. thaliana* seedlings cultured in 6 ml of liquid MSgg of each well of a six-well microplate (Thermo Scientific). Eight seedlings were put in each well. The plant secretions were collected after 4 days, filtered with a 0.22-µm filter, and stored at 4°C for further use.

### Luminescence Experiments

Luminescence reporters were grown in either MSgg medium or MSgg medium containing plant secretions. Experiments were carried using a flat bottom 96-well plate with white opaque walls (Corning). Measurements were performed every 30 min at 30°C for a period for 24 h, using a microplate reader (Synergy 2; BioTek, Winooski, VT, USA). Luciferase activity was normalized to avoid artifacts related to differential cells numbers as RLU/OD.

### Confocal Microscopy

Plants cultured with bacteria were washed in PBS and mounted on a microscope slide and covered with a poly-L-Lysine 31 (Sigma)-treated coverslip. Cells were visualized and photographed using a laser scanning confocal microscope (Zeiss LSM 780) equipped with a high-resolution microscopy Axiocam camera, as required. Data were captured using Zen black software (Zeiss, Oberkochen, Germany).

### Flow Cytometry

Indicated strains used in the experiments were inoculated in 1.5 ml of liquid MSgg without seedlings (control) and MSgg with 14-day-old *A. thaliana* seedlings in a 24-well plate (Thermo Scientific); each well contained eight seedlings. The set was incubated for 24 h in a growth chamber (MRC) at 23°C in a 12-h light/12-h dark regime. After incubation, the seedlings were removed from the liquid medium and washed in PBS for the purpose of removing non-adherent bacteria. Samples were transferred to Eppendorf tube in 500 µl of PBS and vortexed for 1 min, for the purpose of detaching the bacteria from the root. Samples were measured using a BD LSR II flow cytometer (BD Biosciences), using laser excitation of 488 nm, coupled with 505 LP and 525/50 sequential filters. For each sample, 100,000 cells were counted and samples were analyzed using Diva 8 software (BD Biosciences).

### Imaging Flow Cytometry

Samples were prepared as for the flow cytometer. Data were acquired by ImageStream^X^ Mark II (AMNIS, part of Luminex corp., Austin Tx) using a 60× lens (NA = 0.9). Lasers used were 488 nm (200 mW) for GFP excitation and 785 nm (5 mW) for side scatter measurement. During acquisition, bacterial cells were gated according to their area (in square microns) and side scatter, which excluded the calibration beads (that run in the instrument along with the sample). For each sample, 100,000 events were collected. Data were analyzed using IDEAS 6.3 (AMNIS). Single event bacteria were selected according to their area (in square microns) and aspect ratio (width divided by the length of a best-fit ellipse). Focused events were selected by the Gradient RMS and Contrast features (measures the sharpness quality of an image by detecting large changes of pixel values in the image). Cells expressing GFP were selected using the Intensity (the sum of the background subtracted pixel values within the image) and Max Pixel values (the largest value of the background-subtracted pixels) of the GFP channel (Ch02). GFP expression was quantified using the Mean Pixel feature (the mean of the background-subtracted pixels contained in the input mask). The size of bacteria was quantified using the Length feature (measures the longest part of an object, in microns) of the bright-field image.

### Statistical Analysis

All experiments were performed three separate and independent times in triplicates. Datasets were compared using a standard two-way ANOVA, followed by Tukey’s multiple comparison *post hoc* testing, or a pairwise comparison using unpaired *t*-test with Welch’s correction in order to correct for groups with significantly unequal variances. Error bars represented ± SD, unless stated otherwise. *p* < 0.05 was considered statistically significant.

Statistical analyses were performed with GraphPad Prism 9.0 (GraphPad Software, Inc., San Diego, CA).

## Data Availability Statement

The original contributions presented in the study are included in the article/**Supplementary Material**. Further inquiries can be directed to the corresponding authors.

## Author Contributions

HM, OG, IK-G, and ZP designed the experiments. HM, OG, and ZP performed the experiments. HM, ZP, and IK-G analyzed the data. IK-G and ZP wrote the manuscript. All authors contributed to the article and approved the submitted version.

## Funding

The IK-G laboratory is supported by the Israel Science Foundation grant number 119/16 and ISF-JSPS 184/20 and Israel Ministry of Science—Tashtiot (Infrastructures)—123402 in Life Sciences and Biomedical Sciences. IK-G is supported by an internal grant from the Estate of Albert Engleman provided by the Angel–Faivovich Fund for Ecological Research and by a research grant from the Benoziyo Endowment Fund for the Advancement of Science. IK-G is a recipient of the Rowland and Sylvia Career Development Chair.

## Conflict of Interest

The authors declare that the research was conducted in the absence of any commercial or financial relationships that could be construed as a potential conflict of interest.

## Publisher’s Note

All claims expressed in this article are solely those of the authors and do not necessarily represent those of their affiliated organizations, or those of the publisher, the editors and the reviewers. Any product that may be evaluated in this article, or claim that may be made by its manufacturer, is not guaranteed or endorsed by the publisher.

## Supplementary Material

Supplementary Figure S1 Indicated reporter strains for P*_penP_*_-_gfp and P*_ldh_*_-_gfp was analyzed by flow cytometry for **(A)** positively expressing fluorescent populations. Graphs represent mean ± SD or **(B)** the mean intensity of the fluorescent populations. Box and whisker plot shows median and interquartile range, together with the maximum and minimum values and outlier points. Reporter stains were either grown in MSgg medium or in MSgg medium in presence of *A. thaliana* seedlings. Data were collected from 24 h post inoculation, 100,000 cells were counted. Graphs represent results from two independent experiments with n = 2/experiment (total n = 6/group). Statistical analysis was performed using Two-way ANOVA followed by Tukey’s multiple comparison *post hoc* testing. *p* < 0.05 was considered statistically significant.
