# Supplementary figures and images for: Bacillus subtilis Colonization of Arabidopsis thaliana Roots Induces Multiple Biosynthetic Clusters for Antibiotic Production
Source: Front Cell Infect Microbiol. 2021 Sep 3;11:722778. doi: 10.3389/fcimb.2021.722778 (PMC8454505; doi:10.3389/fcimb.2021.722778)

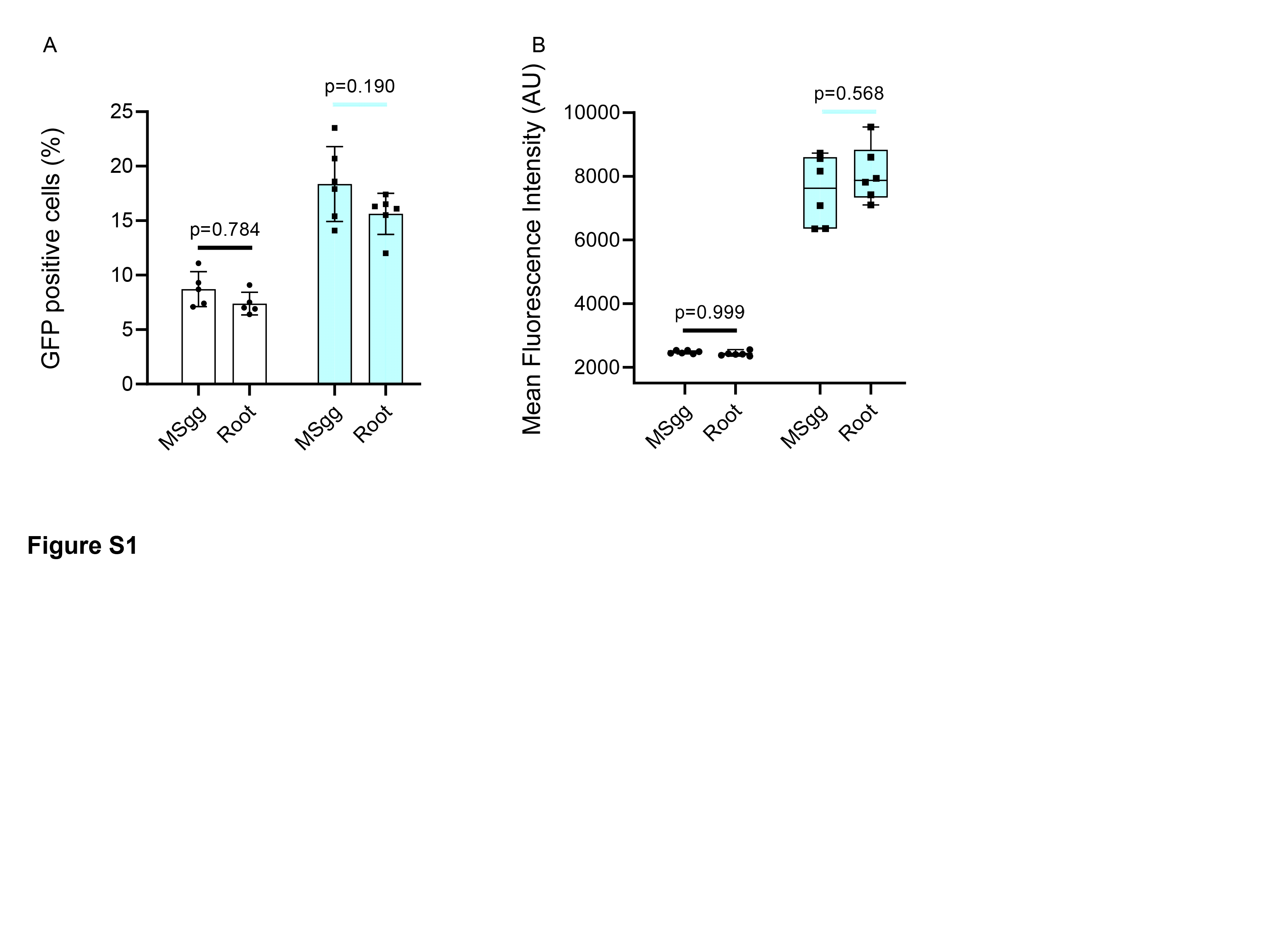

Supplement: Supplementary Figure S1 — Indicated reporter strains for PpenP -gfp and Pldh -gfp was analyzed by flow cytometry for (A) positively expressing fluorescent populations. Graphs represent mean ± SD or (B) the mean intensity of the fluorescent populations. Box and whisker plot shows median and interquartile range, together with the maximum and minimum values and outlier points. Reporter stains were either grown in MSgg medium or in MSgg medium in presence of A. thaliana seedlings. Data were collected from 24 h post inoculation, 100,000 cells were counted. Graphs represent results from two independent experiments with n = 2/experiment (total n = 6/group). Statistical analysis was performed using Two-way ANOVA followed by Tukey’s multiple comparison post hoc testing. p < 0.05 was considered statistically significant. [file Image_1.tif]
